# Early Events Associated with Infection of Epstein-Barr Virus Infection of Primary B-Cells

**DOI:** 10.1371/journal.pone.0007214

**Published:** 2009-09-28

**Authors:** Sabyasachi Halder, Masanao Murakami, Subhash C. Verma, Pankaj Kumar, Fuming Yi, Erle S. Robertson

**Affiliations:** Department of Microbiology and Abramson Comprehensive Cancer Center, Tumor virology Program, University of Pennsylvania School of Medicine, Philadelphia, Pennsylvania, United States of America; Comprehensive AIDS Reseach Center, China

## Abstract

Epstein Barr virus (EBV) is closely associated with the development of a vast number of human cancers. To develop a system for monitoring early cellular and viral events associated with EBV infection a self-recombining BAC containing 172-kb of the Epstein Barr virus genome BAC-EBV designated as MD1 BAC (Chen *et al*., 2005, *J.Virology*) was used to introduce an expression cassette of green fluorescent protein (GFP) by homologous recombination, and the resultant BAC clone, BAC-GFP-EBV was transfected into the HEK 293T epithelial cell line. The resulting recombinant GFP EBV was induced to produce progeny virus by chemical inducer from the stable HEK 293T BAC GFP EBV cell line and the virus was used to immortalize human primary B-cell as monitored by green fluorescence and outgrowth of the primary B cells. The infection, B-cell activation and cell proliferation due to GFP EBV was monitored by the expression of the B-cell surface antigens CD5, CD10, CD19, CD23, CD39, CD40 , CD44 and the intercellular proliferation marker Ki-67 using Flow cytometry. The results show a dramatic increase in Ki-67 which continues to increase by 6–7 days post-infection. Likewise, CD40 signals showed a gradual increase, whereas CD23 signals were increased by 6–12 hours, maximally by 3 days and then decreased. Monitoring the viral gene expression pattern showed an early burst of lytic gene expression. This up-regulation of lytic gene expression prior to latent genes during early infection strongly suggests that EBV infects primary B-cell with an initial burst of lytic gene expression and the resulting progeny virus is competent for infecting new primary B-cells. This process may be critical for establishment of latency prior to cellular transformation. The newly infected primary B-cells can be further analyzed for investigating B cell activation due to EBV infection.

## Introduction

Epstein-Barr virus (EBV) is a ubiquitous human herpesvirus that establishes latent infection in B lymphocytes. EBV is associated with various lymphoid and epithelial malignancies, such as Burkitt's lymphoma, nasopharyngeal carcinoma, lymphoproliferative diseases in immunosuppressed patients, and gastric carcinoma [1] . The principal target cells for EBV infection are human primary B lymphocytes, but the virus can also infect other lymphocytes and epithelial cells [2], [3], [4], [5], [6]. EBV has two alternative lifestyles: latent (non-productive) infection, and lytic (productive) replication [7]. Following primary infection, EBV persists within memory B lymphocytes in a latent state for the life of the host. A low level of reactivation during the lytic cycle allows viral shedding into the saliva and transmission of the virus in vivo [7]. EBV binds to B-lymphocytes through interaction of the glycoprotein gp350/220 with the complement receptor CD21[8]. In vitro, EBV transforms peripheral human B lymphocytes into indefinitely proliferating lymphoblastoid cell lines (LCLs) that allows for genetic manipulation of the virus [7].

Latently infected B cells maintain EBV genomes as 184-kb circular plasmids, referred to as episomes, and express only a limited number of viral gene products [7]. At present, four patterns of EBV latency are recognized [9], [10]. In type I latency, represented mainly by Burkitt lymphoma (BL) cells, viral gene expression is restricted to the two EBER genes, the BART transcripts, and EBNA1 (EBV nuclear antigen 1) [9]. In latency II additional expression of three latent-membrane proteins, LMP-1, LMP-2A and LMP-2B is observed and is most frequently seen in Hodgkin's lymphoma. Latency III is seen in lymphoproliferative diseases developed in immunocompromised individuals and EBV-transformed lymphoblastoid cell lines [7]. In this group all six EBNAs, all three LMPs and the two EBERs are expressed [9], [10]. Type IV latency is less strictly defined and pertains to infectious mononucleosis patients and patients with a post-transplant lymphoproliferative disease [9]. Some individuals also presents with the so called putative latency program (latency 0), in which no detectable latent gene expression is detected [10]. The principal mediators of EBV-induced growth and cellular transformation of B lymphocytes *in vitro* include EBNA2, EBNA3A, 3C and LMP1 proteins [11]. The EBNA genes are important for transformation of primary B lymphocytes, leading to transactivation and regulation of other cellular and viral genes [4], [12]. These proteins are involved in augmentation of the expression of genes coding for CD21, CD23, LMP1 and LMP2 proteins in B lymphocytes [4], [11], [13].

The lytic cascade of Epstein-Barr virus infection is divided into three phases of regulated gene expression, immediate early, early and late [14]. Synthesis of the viral encoded transactivator, BZLF1(also referred to as Zta or ZEBRA) serves as a checkpoint for initiation of the replicative cycle [15]. BZLF1 is a DNA-binding protein, and its expression precedes the switch from latent to lytic infection [4]. BZLF1 is a viral transactivator protein known to be directly involved in triggering expression of the lytic genes and downregulation of latent genes, culminating in cell death and release of infectious virions [15]. This protein up-regulates expression of other immediate early genes as well as its own expression [16]. This immediate early expression in turn up-regulates the expression of early genes such as viral DNA polymerase (BALF5) and thymidine kinase [4], [17]. The major proteins of the lytic phase are the EBV DNA polymerase, BALF5 [18] and the late lytic cascade, major capsid protein, BcLF1[14]. Two small RNAs (EBER-1 and EBER-2) represent the most abundant EBV RNA expressed during latent infection and undergo continuous expression in EBV-positive tumors, independently of the latency type [19], [20].

Conventionally, herpesvirus mutants are generated by homologous recombination in infected cells with DNA fragments or plasmids carrying the mutant allele as described almost 30 years ago [21], [22], [23]. As a consequence, recombination between the herpesvirus genome and the mutant allele gives rise to a mixed population that consists of the wild-type and mutant virus, such that their separation is necessary and important for evaluation of the phenotype. This approach has been proven to be quite tedious with gammaherpesviruses, (i.e. EBV), because so far no host cell type has been shown to fully support the lytic, productive phase of these viruses. In the case of EBV, to study latent genes it is first essential to obtain an immortalized cell line latently infected with the mutant virus, which takes place often in combination with wild-type virus if the gene is essential. To separate these viruses in a second step, the latently infected cell needs to support the lytic phase to produce infectious virions important for establishment of another latently infected, immortalized B cell line exclusively carrying the viral mutant or can be passed into an already immortalized cell line like Ramos or BL41[13]. Because B cell immortalization is a prerequisite to establishment of a mutant EBV LCL, this approach excludes the genetic analysis of genes that are essential for B cell immortalization *in vitro* [24], [25], [26].

The introduction of the bacterial artificial chromosome (BAC) system into the genetics of herpesviruses brought a new dimension to the field [27]. In the BAC system, the entire viral genome can be propagated in *Escherichia coli*, and mutations can be rapidly and precisely introduced into viral genes [27]. To facilitate the generation of recombinant viruses, the EBV genome was first cloned as a bacterial artificial chromosome (BAC). F-plasmid sequences for prokaryotic replication [28], kanamycin resistance marker for prokaryotic selection, and a cytomegalovirus promoter-driven puromycin resistance cassette for eukaryotic selection were inserted into the BamHI site of a plasmid containing EBV BamW DNA and then transfected into B95-8 cells [29]. Following the successful cloning of other herpesviruses, the B95-8 strain of EBV was cloned in a BAC vector [29]. The system employed an epithelial cell background as the virus producing cell and virus production was induced by transfecting an expression vector encoding a viral immediate protein BZLF1 [30], [31] .

In vitro EBV infection results in human B-lymphocytes activation and perpetual proliferation [32], [33]. EBV infected cells grow in tight clumps and express a number of B-cell activation molecules including CD5, CD23, CD39, CD40 and CD44 and proliferation surface antigen marker CD10 as well as intracellular proliferation marker Ki-67 [34]. CD5 expression on B-cell can be up-regulated by various activating agents, which indicate that CD5 is a B-cell activation antigen [35]. CD5 has also been shown to be important for the apoptosis of antigen-receptor induced lymphocytes and for the maintenance of tolerance by B cells [36]. Additionally, previous work reported that CD5 expression is down-regulated by EBV during transformation of CD19 positive B-cells [37]. The B-cell activation marker CD23 has been shown to be upregulated by EBV infection and induced at high level in EBV-transformed lymphocytes [38], [39]. EBNA-2 and LMP-1 cooperatively induces CD23 [13] as well as the human CD40 antigen which is a 50-kD glycosylated phosphoprotein [40]. LMP-1 is a transmembrane protein with a structure reminiscent of G protein coupled receptors, but its signaling activities are similar to that of CD40 which belongs to the TNF receptor family [41]. In addition, depending on the cellular context, LMP1 can induce specific B-cell activation antigens such as CD39, CD23, CD21, CD40 and CD44 [13], [42]. Ki-67 is a monoclonal antibody that recognizes a proliferation related human nuclear antigen expressed during G1, S, and G2/M phase of the cell cycle but not in resting (G0 phase) cells [43], [44].

Gp350 is the glycoprotein found most abundantly on the surface of EB virions as well as on the surface of EBV-infected cells in which EBV is lytically replicating [45]. It is the glycoprotein of the virion that binds to the EBV receptor CD21 (or complement receptor type II, CR2) and initiates infection ([8], [46].The another glycoprotein, gp110 encoded by EBV BALF4 ORF is expressed during the lytic phase of EBV not only at nuclear membrane but also at the cellular membrane and has been shown genetically to be essential for virus maturation [47], [48], [49].

Acyclovir [ACV; 9-(2-hydroxyethoxy methyl) guanine] is one of a class of antiviral compounds effective in curbing a variety of herpesvirus infection in vitro and in animal models [50]. In the EBV system ACV is an effective inhibitor of viral DNA replication in productively infected cells but is essentially devoid of any effect on the replication of viral DNA in latently infected cells, where cellular control mechanisms regulate EBV DNA synthesis[51], [52].

In this report, we generated a recombinant EBV containing a GFP cassette cloned in the BAC vector backbone designated as BAC GFP-EBV in an effort to study the molecular changes during early infection of primary B-cell induced by the virus. [53]. Previous studies by other groups which generated fluorescence tagged EBV proved difficult in our hands [54], [55]. However, the MD1 system generated by Wang and collegues [29] proved to be manipulatable in our hands and so we decided to introduce a fluorescence marker for monitoring infection in this system. The green fluorescent protein is a suitable marker because it fluoresces strongly and stably in mammalian cells and can be monitored by noninvasively strategies in living cells [56], [57]. In this study, a GFP, puromycin and ampicillin cassette was introduced into the EBV- BAC [29] to establish the BAC based recombinant EBV GFP designated as GFP-EBV by homologous recombination, and the recombinant virus DNA was shuttled from *E.coli* to mammalian cells. The induced GFP-EBV virus was used for the infection of Peripheral Blood Mononuclear cells (PBMC) and establishment of lymphoblastoid cell lines (LCLs). Using the GFP-EBV infected PBMCs, we monitored a range of immunophenotypic changes. Several B-cell surface antigen markers such as CD5, CD10, CD19, CD23, CD39, CD40 and CD44 [40], as well as the intercellular proliferation protein Ki-67 were used during initial infection of EBV. We also analyzed the latent and lytic the protein profiles during early infection of primary B-cell by recombinant EBV. Our results suggested that EBV infection to B-cells involves an initial burst of lytic replication which may be critical for the many signaling events involving anticrine and paracrine factors which eventually leads to B-cell transformation and establishment of latency after 2–4 weeks in culture.

## Materials and Methods

### Cells and virus cultures

BJAB was used as EBV negative cell line and LCL1 & LCL2 were used as EBV positive cell lines [58]. BAC-EBV was propagated in EL350 [29] and GFP-Amp cassette was incorporated into BAC-EBV by homologous recombination. BAC GFP-EBV was transferred into HEK 293T cells and the BAC GFP-EBV infected Lymphoblastoid cell lines (LCLs) were established from primary B-cell (Immunology core of UPENN). PBMCs were obtained from UPENN immunology core from de-identified different donors for multiple infection studies. All B-cells were grown in RPMI1640 with 10% fetal bovine serum, and adherent cells in Delbecco's Modified Essential Medium (DMEM) supplemented with 10% fetal bovine serum (FBS), 50 mg/ml streptomycin, and 50U penicillin (medium) (Bio-Whittaker, Walkersville, MD). 1 µg/ml final concentration of Puromycin was used for selection of cells transfected or infected with the BAC GFP-EBV.

### The construction of BAC GFP-EBV genome

For incorporation of GFP in the BAC EBV genome, we considered the region of EBV genome from 149,116 to 154,747 bp. The GFP DNA was introduced into the site of the B95.B deletion at 152,008 and the GFP cassette contained a BamHI site and was flanked by 50 bp downstream and upstream sequence from 152,008 bp [59]. 3646 bp of the GFP cassette (GFP-Puro-Amp) with one BamHI site was electroporated into the bacterial cells EL350 with BAC EBV (MD1BAC) and the cassette was incorporated by homologous recombination where positive clones were screened for ampicillin resistance. The amplicon was transfected into electrocompetent bacterial cells EL350 MD1BAC by electroporation at 1.75 kV. BAC GFP-EBV DNA was extracted from the positive clones and subjected to BamHI digestion overnight. The digested products were resolved on 0.65% agarose gel by running for 16 h–20 h at 40V and visualized by ethidium bromide staining and UV exposure. The digestion pattern was analyzed and compared with wild type MD1BAC. The resolved gel was transferred onto the Genescreen tranfer membrane (PerkinElmer, Waltham, MA, USA) and the cassette was visualized by hybridizing with the EBV BamHI I fragment as a probe.

### Transformation of recombinant BAC GFP-EBV into bacteria

Oligonucleotide primers corresponding to the different region of EBV genome were synthesized 5′ to 3′. The sequence of oligonucleotides and the product length are shown in table 1.

**Table 1:** Primers used in PCR analysis[59].

Primers	Product size	Oligonucleotide sequence	EBV genome
BamHIT sense	350 bp	5′-CCCCCTTTTCCGCATCAG-3′	140112-140461 bp
BamHIT antisense		5′-AGTCCGGATTGGGCACCA-3′	
BamHIK sense	341 bp	5′-GCTGCTTTCCTCGGATGCC-3′	112231-112571 bp
BamHIK antisense		5′-CTGGGATGGGGAGCGGAG-3′	
BamHI E sense	1128 bp	5′-TACTGCCACCAGTACCACAACA-3′	97001-98128 bp
BamHI E antisense		5′-GGCCGACATTCTCCAAGATAA-3′	
BamHI H sense	194 bp	5′-CTCTGCCACCTGCAACACTA-3′	49117-49310 bp
BamHI H antisense		5′-ATTTGGGGTGCTTTGATGAG-3′	
Fragment C sense:	761 bp	5′-GCAGGGCTCGCAAAGTATAG-3′	11095-11855 bp
Fragment C antisense		5′-TGCGGAAGTGACACCAAATA-3′	
Puro sense	220 bp	5′-CGTGCAGTGCTTCAGCCGCTACCCC-3′	152856-153075 bp
Puro antisense		5′-CTTGTGCCCCAGGATGTTGCCGTCC-3′	
EGFP Sense	552 bp	5′-GACGTAAACGGCCACAAGTT-3′	152716-153267 bp
EGFP antisense		5′-CTGGGTGCTCAGGTAGTGGT-3′	
E3Ct1t2 sense	152 bp	5′-AGAAGGGGAGCGTGTGTTGT-3′	99939-100091 bp
E3Ct1t2 antisense		5′-GGCTCGTTTTTGACGTCGGC-3′	
EBVGFP sense	820 bp	5′-GGGCTCGTTTAAACAAAGTCTCATC-3′	151921-152740 bp
EBVGFP antisense		5′-CGCTGAACTTGTGGCCGTTTACGTC-3′	

The genomic DNA was prepared from approximately 5×10^4^ cells. Following centrifugation to remove medium, cells were resuspended in 0.2× phosphate buffered saline, boiled for 10 min, and mixed with 0.1 volume of 10-mg/ml proteinase K (Sigma, Marlborough, MA), and the mixture was incubated for 30 min at 55°C. Proteinase K was inactivated by incubation at 95°C for 20 min. PCR analysis was performed by using Perkin-Elmer thermal cycler with 5 µl of DNA in a 50 µl reaction. PCR-amplified DNA was analyzed by electrophoresis using 2% agarose gels and visualized by staining with ethidium bromide and UV exposure.

### Tranfection of GFP BAC-EBV into 293T cells

HEK293T cell were transfected with 5 to 10 µg of BAC GFP-EBV DNA by lipofectamine 2000 (Invitrogen, Inc., Carlsbad, CA) according to manufacturer instruction. After 2 days post transfection, cells were trypsinised and plated at 10^4^ cells per well in 96-well tissue culture plates in DMEM medium and the medium was replaced with fresh 1 µg/ml puromycin-containing DMEM medium every three days. Puromycin-resistant clones (shown in Table 2) were screened for the presence of episomally maintained BAC GFP-EBV by visualization of GFP protein.

**Table 2:** No. of clones generated in HEK293T and LCLs of BAC GFP-EBV.

Cell	No. of clones
293T	4,10,11,12,15
BAC GFP-EBV	
LCL	10,11,14,15
BAC GFP-EBV	

### Induction of virus from 293T cells containing GFP EBV BACmid

Cells harboring BAC GFP-EBV were induced to release virus by culturing for 5 days in complete RPMI 1640 medium containing phorbol ester TPA (12-O-tetradecanoylphorbol-13-acetate, 20 ng/ml) and butyric acid (BA, 3 mM; both from Sigma). Cell suspensions were centrifuged at 1,800 rpm for 10 min and the supernatant was filtered through a 0.45 micron cellulose acetate filter. The viral particle were concentrated by ultracentrifugation at 27K rpm at 4°C and stored at −80°C.

### Infection of primary human B cells with GFP-EBV

Lymphoblastoid cell lines were generated by infections of 1×10^6^ primary B-cells incubating with virus suspension in 1 ml of RPMI 1640 (10% Fatal Bovine Serum) medium in the presence of cyclosporin A (Sigma, Marlborough, MA) and incubated for 4 hrs in 37°C. Cells were centrifuged for 5 min at 1500 rpm, the supernatant discarded, pelleted cells were resuspended in fresh RPMI 1640 (10% FBS) medium in 96 well plates. The infection was checked by the visualization of GFP expression using fluourescence microscopy. The transfected green cells were enriched by selection with puromycin. The infected cells were then transferred to 48 well plates and expanded to larger well plates until the cultures continued to grow continuously in complete media. The number of clones generated for BAC GFP-EBV LCL was also shown in table 2. To monitor the early stage of infection after 4 hrs incubation with EBV and PBMC, the cells were then washed two times with fresh RPMI 1640 (10% FBS) medium to remove excess virus and fresh medium was added. This infection step is designated as Infection I. The supernatant from primary infection (Infection I) of 2×10^7^ PBMC by GFP-EBV were used infection of fresh PBMC (1×10^6^) in a similar manner and infection further monitored by GFP fluorescence. This step of infection was designated as Infection II. Infection I was also monitored by adding 25 µM acyclovir and the supernatant from infection I were also used for infection II to determine if virus produced was due to replication during lytic replication during lytic infection in Infection I and not due to virus passed on from the initial infection .

### Western Blotting

Cell lysates were electrophoresed on SDS-PAGE gels and transferred to 0.45 micron nitrocellulose membranes. Blots were then probed using specific primary antibodies (S12) for LMP-1 [60] and human serum (KJ) for EBNA-1 and A10 for EBNA-3C [61] with required dilutions. This was followed by incubation with fluorescence labeled secondary antibodies, Alexa Fluor 680 and Alexa Fluor 800 (Molecular Probes Inc., Carlsbad, CA; and Rockland Inc., Gilbertsville, PA, respectively) diluted at 1∶20,000. Blots were visualized and analyzed using LICOR Odyssey imaging system and Odyssey software (Li-Cor, Lincoln, NE).

### Flow cytometry analysis of EBV infected peripheral blood mononuclear cells

Peripheral Blood Mononuclear Cells (PBMCs) (procured from Immuology core, University of Pennsylvania Medical School, Philadelphia, PA) were infected with BAC GFP- EBV and the infected cells were fixed with 0.5% paraformaldehyde at 6 h, 12 h, 24 h, 48 h, 72 h, 96 h, 120 h and 168 h post-infection for 1 hour at 4°C and washed three times with buffer W (1X PBS with 0.1% BSA and 0.001% NaN3). A broad panel of fluorochrome-conjugated monoclonal antibodies was used for detection of the following cell surface markers: CD3, CD5, CD10, CD19, CD23, CD39, CD40, CD44 and Ki-67, respectively. The surface antigen markers contained different conjugates such as Phycoerythrin (PE), Phycoerythrin-Cy7 (PE-Cy7), Per CP, PercpCy 5.5 and Allophycocyanin (APC).

Flow cytometry was carried out on an 8-color flow cytometry instrument CYANADP (Wistar Institute, Philadelphia, PA) with Cell-Quest software (Becton-Dickinson, San Jose, CA) used in accordance with the manufacturer's instructions. Instrument settings were adjusted so that fluorescence of cells from uninfected controls, in the case of GFP readings, or negative controls (i.e., with antibody omitted in antibody labeling) fell within the first decade of a four decade logarithmic scale on which emission is displayed. Flow cytometry plots showed at least 20000 events. The data were analyzed by FlowJo software (Becton-Dickinson, San Jose, CA). The expression levels of different surface antigen markers as well as an intracellular proliferating marker were analyzed from GFP positive EBV infected cells. The extent of infected B-cell and T-cell population from total cell populations were analyzed from CD19 and CD3 positive cells.

### Real time PCR

Total RNA were prepared from 5×10^6^ GFP EBV infected PBMCs after different times post-infection (6h, 12h, 24 h, 48h, 72h, 96h, 120h and 168 hours) using Trizol reagent (Invitrogen, Inc., Carlsbad, CA) according to manufacturer's instructions. cDNA was synthesized using a Superscript II RT kit (Invitrogen, Inc., Carlsbad, CA) according to the manufacturer's instructions. The specific primers used for the amplification of latent genes (EBNA-1, EBNA-2, LMP-1), lytic genes (BZLF1, BcLF1 and BALF5) and internal control GAPDH are shown in Table 3. The lytic gene, BALF5, was also amplified in the presence of 25 µM ACV.

**Table 3:** Primers used in q-Real time PCR[59].

Primer Name	DNA Sequence (5′-3′)	Product length
EBNA-1	5′-CATTGAGTCGTCTCCCCTTTGGAAT-3′	150 bp
	5′-TCATAACAAGGTCCTTAATCGCATC-3′	
EBNA-2	5′-GAGACCAGAGCCAAACACCTCCAGT-3′	150 bp
	5′-TTAGGGGTTGCCGTGTGTGAATTTC-3′	
LMP-1	5′-CCCGCACCCTCAACAAGCTACCGAT-3′	150 bp
	5′-TTGTCAGGACCACCTCCAGGTGCGC-3′	
BZLF1	5′-AACCGCTCCGACTGGGTCGTGGTTT-3′	150 bp
	5′-CCAGGTTGAGGTGCTTCTCCCCCGG-3′	
BcLF1	5′-CCTCTTGGAATGCAGCTGGGGCCAG-3′	150 bp
	5′-CCAATTATGACCTGCTGCGGCTGGA-3′	
BALF5	5′-GCTGGCCTTGAGGGCGCTGAGGACT-3′	259 bp
	5′-CACCCACGGAAGCCCTCTGGACTTC-3′	

The target gene was amplified from cDNA using SYBR green real-time master mix (MJ Research Inc.,Waltham,MA), 1 mM each primer and 5 µl of the cDNA product in a total volume of 20 µl. Thirty-five cycles of 1 min at 94°C, 1 min at 55°C and 1 min at 72°C, followed by 10 min at 72°C, were performed in an MJ Research Opticon II thermocycler (MJ Research Inc., Waltham, MA). Each cycle was followed by two plate reads, with the first at 72°C and the second at 85°C. A melting curve analysis was performed to verify the specificity of the amplified products, and the values for the relative quantitation were calculated by the ΔΔCt method [62]. All experiments were performed in triplicate.

### Immunofluorescence

GFP infected PBMC cells after different time intervals of postinfection were dried onto slides and fixed using a 1∶1 mixture of acetone and methanol. After fixation cells were extensively washed in PBS and incubated in blocking buffer [PBS supplemented with 0.1% Triton-X 100, 0.2% fish skin gelatin (Sigma)] at room temperature for 30 min. Endogenous expression of gp350 and gp110 were detected using mouse monoclonal antibody (1∶500 dilution), and rabbit (1∶250 dilution) respectively. Primary antibodies were diluted in blocking buffer and incubated with fixed cells for 1 h at RT. Slides were washed three times (5 min each) with PBS and incubated with appropriate secondary antibody (1∶2000) for 1 h at RT followed by three times washes (5 min each) with PBS. The last wash contained 4′, 6′-diamidino-2-phenylindole (DAPI; Promega Inc., Madison, WI) for nuclear staining. Goat anti-mouse antibody Alexa Fluor 594 and goat anti-rabbit antibody Alexa Fluor 594 were purchased from Molecular Probes Inc.(Carlsbad, CA). Slides were then washed in PBS and mounted using Prolong anti-fade (Molecular Probes Inc, Carlsbad, CA). Fluorescence was viewed by confocal microscopy and analyzed with Fluoview 300 software from Olympus Inc. (Melville, NY).

## Results

### Incorporation of GFP into BAC EBV (MD1BAC) and selection in mammalian epithelial cells

**Figure 1:**
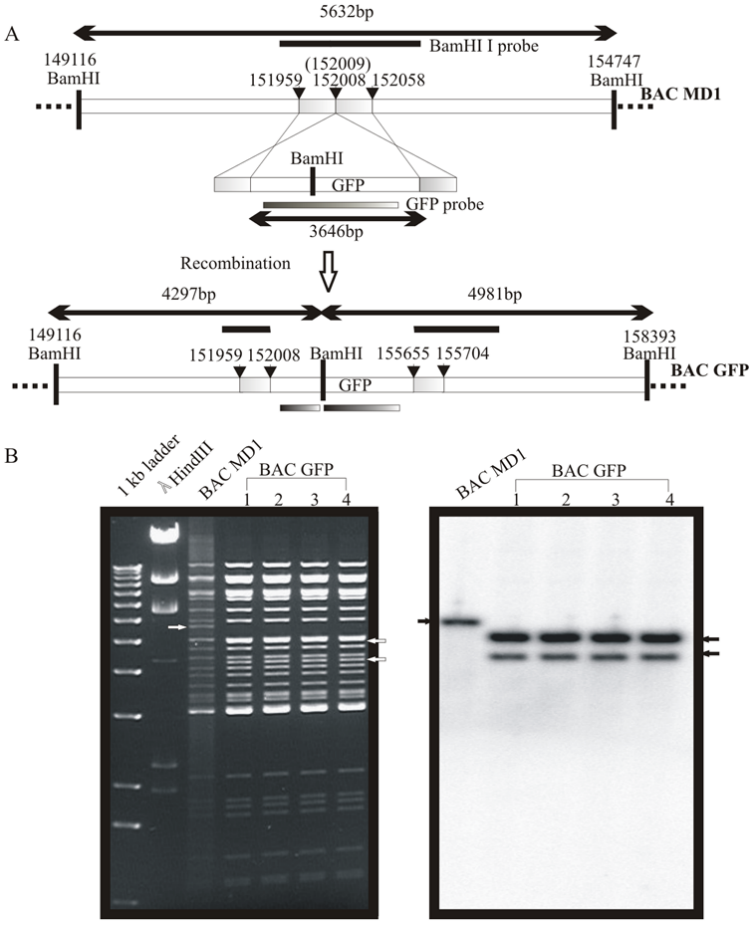
Strategy for insertion of the GFP cassette into BAC EBV (MD1BAC). (A) BamHI region of EBV genome (149116 bp -154747) [59] was targeted for homologous recombination. A DNA fragment was generated by PCR amplification of a GFP/AMP cassette with a BamHI site using primers incorporating 50 nucleotides of the EBV genome upstream of the 152008 nucleotide and downstream of the 152009 nucleotide at the 5′ termini. The fragment was electroporated into E.*coli* EL350 carrying EBV bacmid (MD1BAC) and expressing recombinase to allow homologous recombination. After recombination the BamHI site splits to two fragments compared to BACMD1. (B) The BamHI digestion pattern of BAC GFP-EBV and MD1BAC was visualised in 0.65% agarose (left panel). The fragments which split in to two fragments are indicated by white arrows. BamHI I fragment was used as a probe for southern blot analysis (left panel). The southern blot analysis showed that the 5.6 bp fragment from MD1BAC (white arrow) and two BAC GFP-EBV fragments ∼5 kb and ∼4.3 kb.

To incorporate the GFP cassette into the BAC vector sequences containing the entire EBV genome, designated as MD1BAC [29], we transfected a GFP cassette which contained GFP, puromycin and ampicillin resistance genes under the control of the CMV promoter into EL350 by homologous recombination between the viral genome (Fig. 1A). Ampicillin was introduced along with the GFP cassette into EBV genome with the BAC vector backbone for selection in *E.coli* [29]. Viral genome position 152,008 was chosen for insertion of the cassette as there is no identifiable open reading frame from 151,959 to 152,058 i.e. 100 bp is unique sequence in B95-B EBV genome [59].

**Figure 2:**
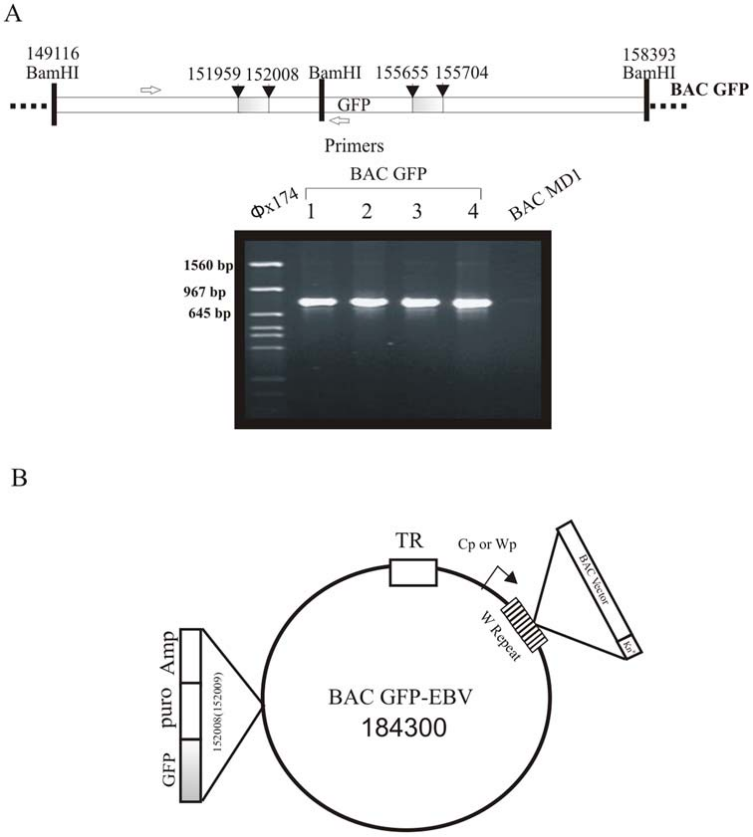
Verification of recombinant BAC GFP-EBV. (A) The DNA from bacterial colonies screened by kanamycin-ampicillin together and sequence analysis of the inserted GFP/amp into MD1BAC genome by PCR amplification, with one primer of EBV genome and another primer from GFP. Amplification of the 820 bp fragment was shown from DNA observed from 4 independent isolates (2–5 lanes) with MD1BAC as negative control. (B) Schematic of EBV showing position of the markers and GFP.

The GFP cassette was amplified from GFP-pBSpuro cassette [63] with 50 nucleotides of EBV genome (151,959-152,008 and 152,008-152,058) at either terminal of the cassette (Figure 1A); the amplified cassette was then transfected in EL350 with MD1BAC for homologous recombination and the clones were screened on amp-kan plate (kanamycin was already inserted into BAC EBV in MD1BAC [29]). The DNA was extracted from amp-kan resistant EL350 colonies and digested with BamHI. The digestion pattern of BAC GFP-EBV is shown in Figure 1B (left panel). The GFP cassette had a BamHI site, therefore the BamHI fragment containing the 152,008 site split into two fragments of 4,981 bp and 4,297 bp after BamHI digestion (Figure 1B, arrow heads). The digestion profile compared with MD1BAC clearly indicated that there were full EBV genomes with the GFP cassette incorporated at the desired site (Figure 1B, left panel). Generation of two bands after cassette introduction was confirmed by Southern blot analysis using the BamHI I fragment as a probe (Figure 1B, right panel). Inclusion of the GFP cassette at this site was also confirmed by amplifying the junction region of EBV gene and GFP cassette. The amplification of an 820 bp size PCR product, which is shown in Figure 2, is identical with the predicted size shown in schematic diagram. The GFP incorporated MD1BAC was then designated as BAC GFP-EBV.

**Figure 3:**
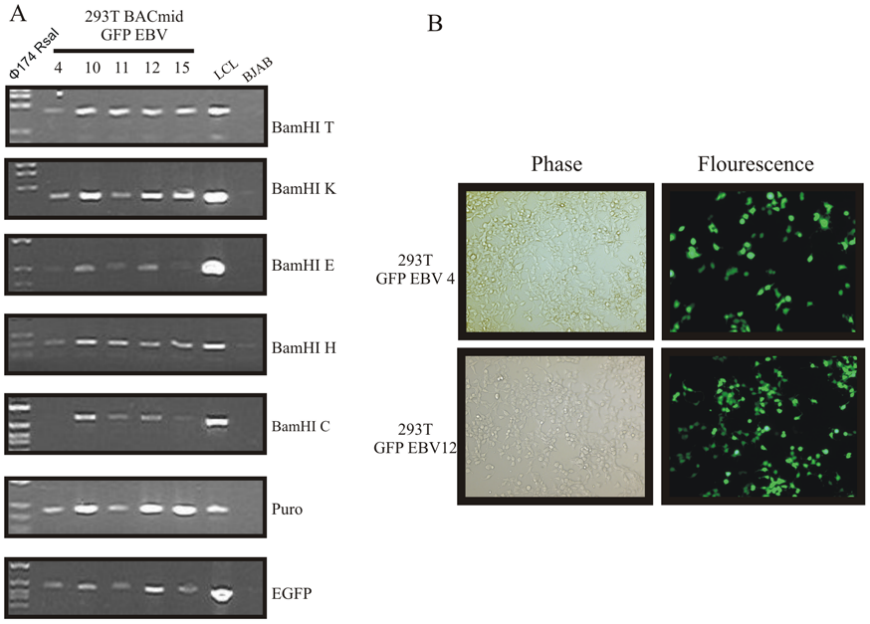
Transfection of Full length BAC GFP-EBV into 293T cells. (A) BAC GFP-EBV DNA was transfected to 293T and stable cell line generated as screened by puromycin. Different genes encoding by EBV (BamHI T, BamHI K, BamHI E, BamHI H and BamHI C) as well as puromycin and GFP were analyzed by PCR amplification taking 5 different transfected clones (4, 10, 11, 12 and 15). BJAB and LCL were taken as negative and positive control, respectively. (B) Phase contrast (left panel) and Flourescence images (right panel) showed two indifferent (number 4 and 12) containing BAC GFP-EBV transfected into 293T stable cell line screened by puromycin.

To test whether the BAC GFP-EBV clone was competent for viral replication and B-cell immortalization, the BAC GFP-EBV was induced for replication in 293T cells [29], [54]. Highly pure BAC GFP-EBV DNA was prepared and transfected into HEK 293T cell. Two days after transfection, 10–20% of the cells typically showed GFP expression. Transfected cells were selected by puromycin in 96 well plates. After Four to six weeks of puromycin selection, 5 stable HEK 293T clones of BAC GFP-EBV expressing GFP were selected for further analysis. The GFP signals for two such clones are shown in Figure 3B. Persistence of the full length EBV genome was determined by PCR analysis of hirt extracted DNA from these stable cell lines using 5 primer sets across the entire genome (Figure 3A). The PCR results suggested the presence of full length intact EBV containing GFP and Puromycin markers within the BAC GFP-EBV. Amplification of the different regions across the EBV genome confirmed that these stable cells were able to maintain the intact BAC GFP-EBV.

### Infection of primary B-cell with BAC GFP-EBV and establishment of LCLs

**Figure 4:**
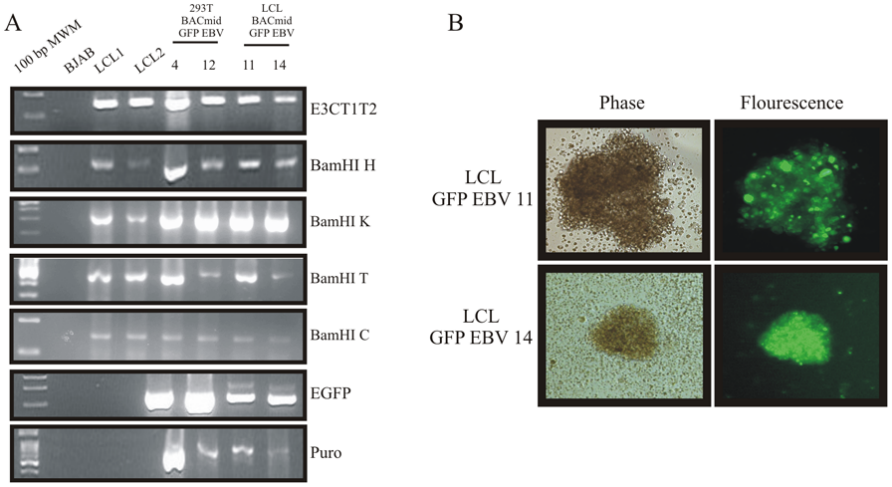
LCLs established by infection with GFP-EBV. (A) PBMC cells were infected by GFP- EBV and made GFP positive LCLs selected with puromycin. The proliferating cells are clustered and GFP positive. BAC GFP-EBV transfected 293T cell clones and LCL established EBV encoded different region of EBV (E3CT1T2, BamHI H, BamHI K, BamHI T and BamHI C) as well as puromycin and GFP were checked by PCR amplification taking 2 different infected LCL clones (11 and 14). BJAB was used as negative control and LCL1 & LCL2 were used as positive controls, respectively. (B) Phase-contrast (left) and fluorescence (right) images of 2 different established (11 and 14) GFP-EBV LCLs.

BAC GFP-EBV virus from the stable 293T cells were induced by treating with chemical inducers, TPA and butyric acid for 4 days [29]. The supernatant containing GFP EBV was used to infect PBMC after concentrating by ultracentrifugation. Human primary B-cells were infected by using different volumes of the concentrated virus. The infected cells were incubated at 37°C and grown overnight. The cells having high GFP expression and B-cell clumping were then diluted and plated into 96 well plates and incubated for 3–4 weeks. The infection was monitored by GFP expression. Approximately, 20–30% of the cells were positive for green flourescence after 36 hours of infection. Four weeks later, these green cells were clearly transformed to LCLs. The GFP content in LCL was enhanced by puromycin screening, and stable LCLs with GFP expression were obtained from the above pool of cells as shown in Figure 4B. To monitor whether or not the GFP-EBV positive LCLs were intact, the genome was analyzed by PCR amplification across different regions of the EBV genome (Figure 4A). PCR analysis of the different region of the EBV genome in LCLs suggested that the LCL maintained an intact EBV genome along with GFP cassette (Figure 4A).

### Analysis of EBV latent proteins expression in GFP-EBV positive LCLs

**Figure 5:**
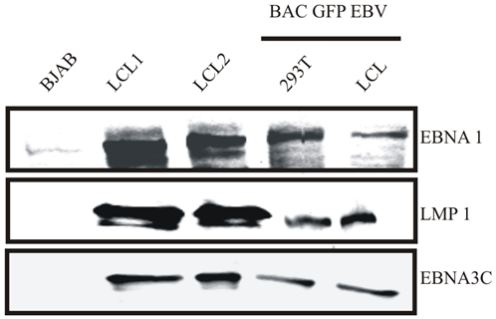
Latent gene expression. Latent proteins expressions were analyzed in cells infected with in BAC GFP-EBV stably transfected 293T and LCLs made from BAC GFP-EBV infection. Expression of EBV EBNA-1, EBNA-3C and LMP-1 were determined by Western blotting.

In latency type III, six nuclear antigens (EBNA1, EBNA2, EBNA3A, EBNA3B, EBNA3C, and EBNA-LP), three latent membrane proteins (LMP1, LMP2A, and LMP2B), two small nonpolyadenylated RNAs (EBER-1 and EBER-2), and transcripts from the BamHI-A region (BARTs) are expressed [64]. To further investigate the establishment of latent infection due to GFP-EBV, the latent protein expression profile of the critical latent antigens in these GFP-EBV transformed LCLs were analysed. The protein expression in HEK 293T containig BAC GFP-EBV and GFP-EBV infected LCLs were analysed by immunoblotting along with BJAB as negative control and previously created LCLs as a positive control (Figure 5). Antibodies detecting EBNA3C and EBNA1 and the oncoprotein LMP-1 were used to detect the specific antigens. The expression patterns of these proteins are shown in Figure 5. Detection of EBNA-1, LMP-1 and EBNA-3 proteins clearly showed that the recombinant GFP-EBV was capable of transforming primary B-cells into LCLs and was also able to maintain a type III latency program.

### EBV infection induces B-cell activation and cell proliferation

To investigate the immunophenotypic effects of EBV infection on B-cell during early infection, we analyzed the expression profile of several B-cell activation and proliferation surface antigen markers (CD5, CD10, CD23, CD39, CD40, and CD44) as well as the intracellular proliferation marker (Ki-67) at different times post-infection.

**Figure 6:**
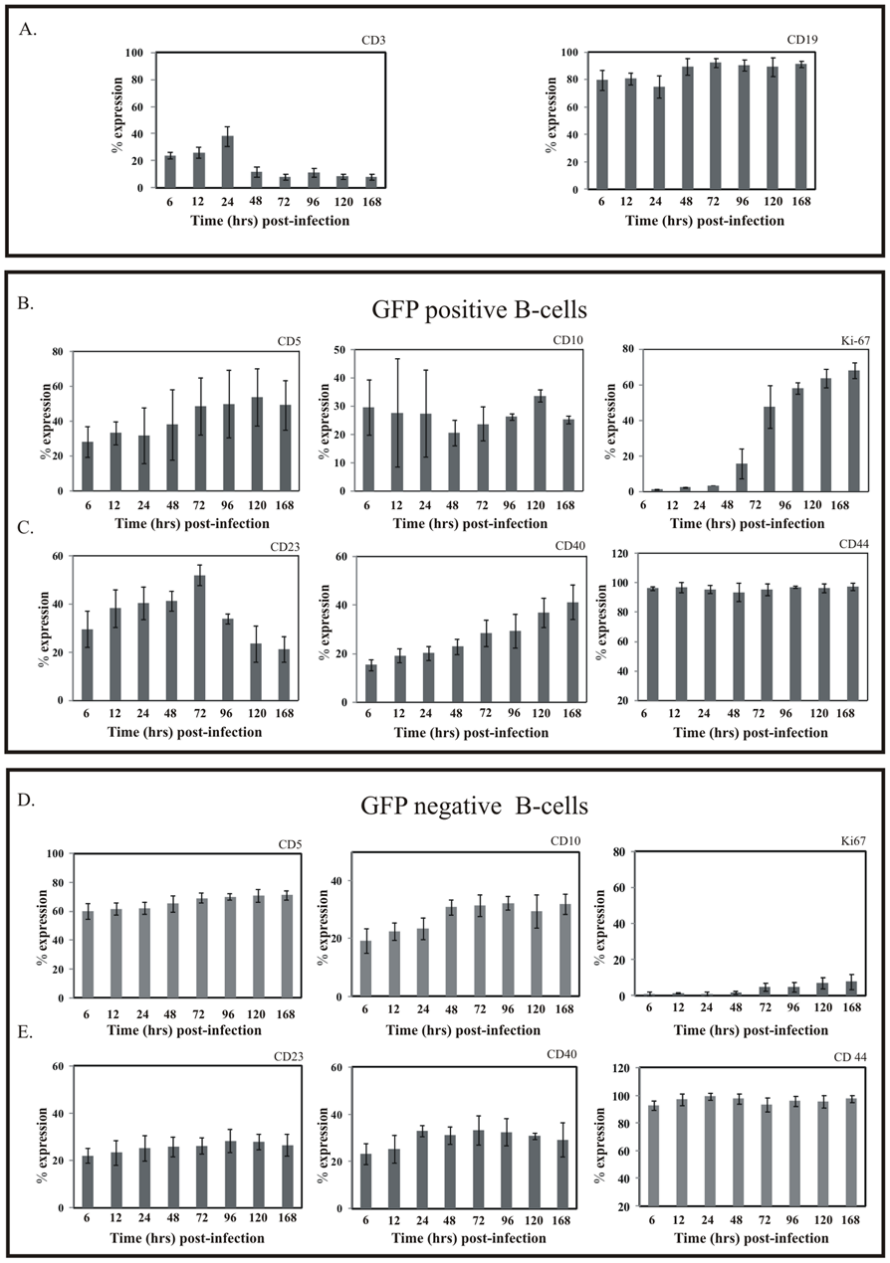
Expression of B-cell proliferation and activation markers due to GFP-EBV early infection. 2×10^6^ PBMC were infected by GFP-EBV and (A) describe CD3 and CD19 GFP population. (B) the expression of proliferation markers (CD5, CD10 and Ki-67) and (C) activation markers (CD23, CD40 and CD44) were measured by Flow cytometry after different time post-infection (6h, 12h, 24h, 48h, 96h, 120h and 168h) with GFP-EBV infected cells (i.e. GFP-positive cells). (D) The expression of proliferation markers (CD5, CD10 and Ki-67) and (E) activation markers (CD40, CD44 and CD23) were measured by Flow cytometry after different time post-infection (6 h, 12 h, 24 h, 48 h, 96 h, 120 h and 168 h) with GFP-EBV non-infected B-cells (i.e. GFP-negative B-cells).

Since EBV also infects T-cells along with B-cell, we investigated EBV infected T-cell population by analyzing the expression of CD3 antigen at different times postinfection. The expression of CD3 in GFP positive cells showed that 20% of the CD3 positive T cells were positive by 6 hours infection increasing to 38% by 24 hours. However, this signal was dramically decreased by 48 hours and went down further to 8% after 168 hrs (Figure 6A, left panel). Of significance, the extent of B-cell infectivity measured by GFP positive CD19 expression was very high within 6 hours postinfection increasing to above 90% after 7 days postinfection (Figure 6A, right panel).

PBMCs were infected by GFP-EBV and the infected cells were subjected to flow cytometry analysis at different times post-infection. The expression of the indicated surface antigen markers were determined up to 7 days for GFP positive EBV infected cells.

The expression of the CD5 cell surface marker did show a gradual change during the course of early infection. By 6 hours post-infection only 28% of the GFP positive cells were expressing CD5 (Figure 6B). This was increased to 54% by 5 days later. Interestingly the percent of GFP positive cells that were CD5 positive were dramatically less by 6–7 days suggesting that this initial increase was not sustained after 5 days with development of LCLs (Figure 6A, left panel). However, the CD10 activation marker showed that about 30% of the GFP-positive cells expressed CD10 by 6 hours postinfection and this was relatively unchanged by 7 days postinfection (Figure 6A, middle panel). The expression pattern of CD23 during early infection with GFP-EBV was interesting with an increase in the percentage of GFP positive cells about 30% by 6 hours postinfection increasing to about 50% by 72 hours post-infection and then showed a rapid decline immediately after which continued to 7 days later (Figure 6C, left panel). Importantly, a well-known B-cell activation and proliferation marker CD40 was detected in about 15% of the GFP-positive cells and gradually increased to over 50% by 7 days postinfection (Figure 6C, midle panel). The expression of CD44 was unchanged throughout the events of early infection (Figure 6C, right panel). However, the percentage of EBV positive cells as determined by GFP expressing CD39 was significant, approximately 90%, but gradually decreased to 42% by 7 days postinfection (data not shown).

To study proliferation due to infection of primary B-cell by GFP-EBV on B-cell, we monitored expression of the intracellular proliferation marker Ki-67 [34] by Flow Cytometry. Ki-67 was expressed at 48 hrs of post-infection of GFP EBV and its signal was consistently increased up to 7 days post-infection (Figure 6B, right panel). Importantly, analysis of LCLs showed extremely high levels of Ki-67 suggesting a requirement for Ki-67, or that it has a critical role in initiating and maintaining the transformed state induced by the virus.

For further investigation of the immunophenotypic effects of EBV infection on B-cell during early infection, we also analyzed the expression profiles of several B-cell activation and proliferation surface antigen markers (CD5, CD10, CD23, CD39, CD40, and CD44) as well as the intracellular proliferation marker (Ki-67) at different times post-infection in non-infected B-cells i.e. GFP negative cells. Flowcytometry analysis from GFP-negative cells showed that there was no significant change in surface antigen markers (CD5, CD10, CD23, CD40 and CD44) or the intercellular marker Ki-67 during early infection as shown in figure 6D and figure 6E.

### Rapid induction of EBV lytic replication during early infection of human primary B-cells

**Figure 7:**
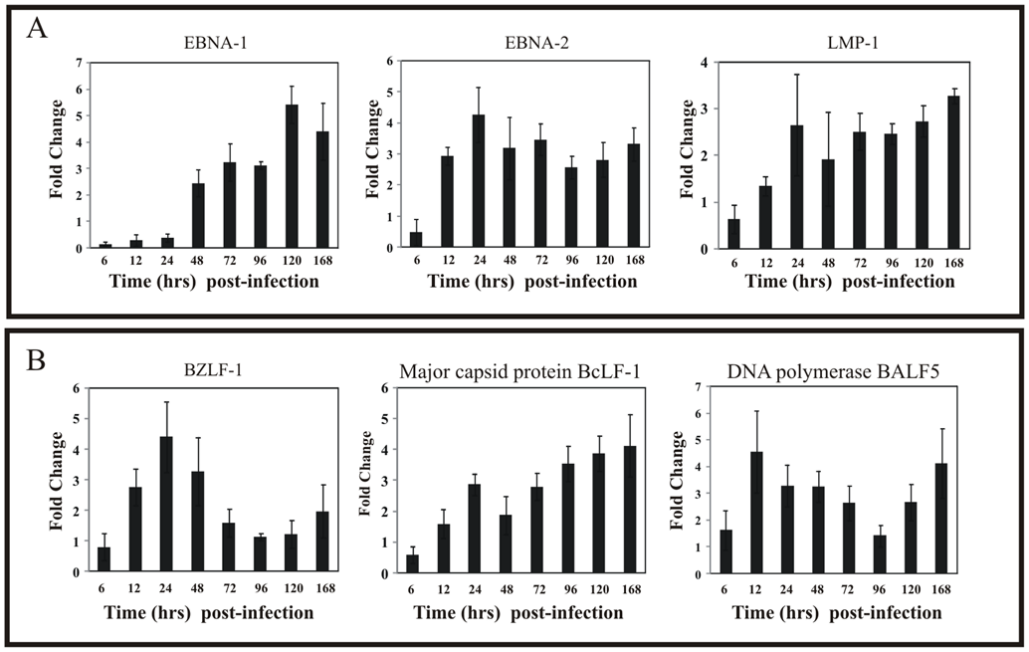
Latent and lytic gene expression during GFP-EBV early infection. 5×10^6^ of PBMC cells were infected with GFP-EBV. (A) The designated time of GFP-EBV postinfection (6h, 12h, 24h, 48h, 72h, 96h, 120h and 168h) of early infection, the expression of latent genes EBNA-1, EBNA-2 and LMP-1 mRNAs were examined by qReal Time PCR. (B) The lytic genes mRNAs BZLF1 which is the immediate early transcriptional and replication protein, major capsid protein BcLF1 and DNA polymerase BALF5 mRNA were also examined by qReal Time PCR after GFP-EBV infection at similiar intervals stated above. To determine quality of the RNA, GAPDH mRNA was also amplified by RT-PCR. The fold change was calculated by the ΔΔCt method. Each data point shown is the average of three identical experiments. ± SD was shown in error bar.

EBV establishes different types of latency characterized by differential expression of a group of latency proteins [14]. To evaluate the gene expression pattern during early events of infection, GFP-EBV was used to infect PBMCs and latent and lytic gene expression were analyzed. Latent expression of EBNA-1, EBNA-2 and LMP-1 and the lytic genes immediate early transactivator (BZLF1), major capsid protein (BcLF-1) and DNA polymerase (BALF5) were monitored. The transcript levels of these genes were determined from the total RNA extracted from GFP-EBV infected PBMCs at different time points post-infection up to 7 days (Figure 7).

The copies of latent gene transcripts at different time points post-infection of GFP-EBV was determined by semi-quantitative real time PCR as shown in Figure 7A. Expression of EBNA-1 was barely detectable at 24 hrs, but was clearly detected at 48 hrs and peaked by 120 hrs. EBNA2 signals were evident by 6 hrs and plateaued by 24 hrs post-infection (Figure 7A, middle panel). EBNA-2 transcript was consistently detected through 7 days of infection at similar levels up to 7 days throughout the course of the study. Similarly, LMP-1 transcript levels was detectable at 6 h and reached at maximum level by 24 hours of post-infection (Figure 7A, right panel). Interestingly, the level of LMP-1 transcripts remained consistent up to 7 days post infection.

The mRNA levels of lytic genes BZLF1, BcLF1 and BALF5 were also determined using real time qPCR at different time points post infection. We found that the BZLF-1 gene was expressed during the initial stages of infection i.e. it came on at 6 hrs post-infection and peaked at 24 hrs (Figure 7B, left panel). Interestingly, the BZLF1 transcripts decreased after 24 hrs but began to increase again by 96–120 hours post-infection. Another lytic gene BcLF-1 was detected at 6 hrs post-infection and continued to increase throughout the course of study. The expression of DNA polymerase (BALF5) during early infection peaked by 12 hours post-infection followed by a gradual reduction to 96 hrs. However, the level of BALF5 was also increased again by 7 days (Figure 7B). Interestingly, the levels of lytic transcripts as determined by Real time PCR was greater than that compared to the latent genes during early stage of B-cell infection by GFP-EBV.

### The progeny GFP-EBV produced during early infection is infectious

**Figure 8:**
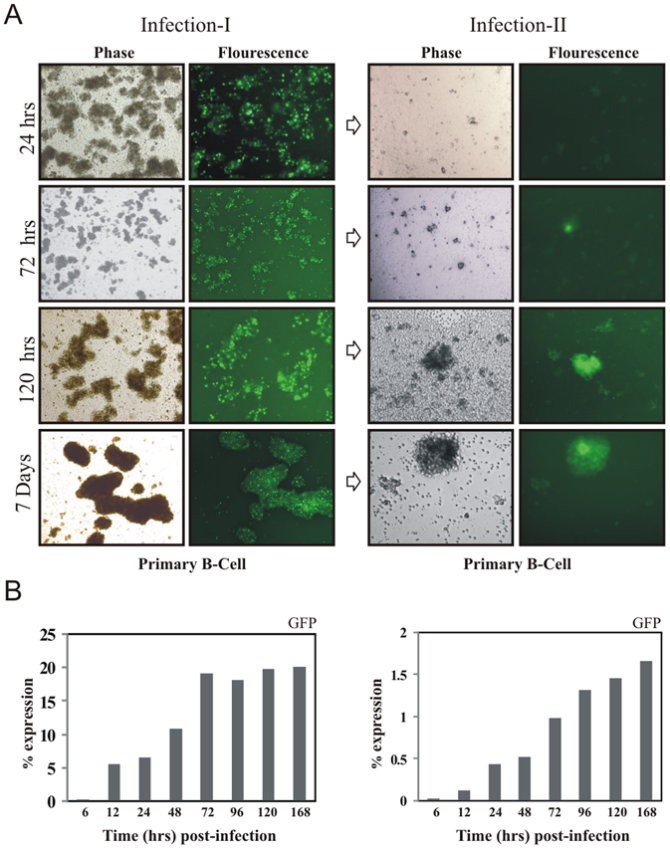
Lytic burst of EBV virus during early infection of GFP-EBV. PBMC cells were infected with GFP-EBV and at specific times postinfection the supernanat was collected and used infect fresh PBMC cells. (A) Phase-contrast (left) and fluorescence (right) images of GFP-EBV infected PBMC cells are shown after specific times postinfection (24 hrs, 72 hrs, 120 hrs and 168 hours) (left panel). Phase-contrast (left) and fluorescence (right) images of PBMC cells infected with supernatant from the above mentioned times post-infection (from 24hrs, 72hrs, 120hrs and 168h) (right panel). B. Flow cytometry analysis of GFP expression at post-infection of different time intervals (6h, 12h, 24h, 48h, 72h, 96h, 120h and 168h) (left panel), and after infection of fresh PBMC from supernatant of infected cells (right panel).

To investigate further our hypothesis that early lytic infection produces viral progeny capable of infecting new cells, we used supernatant collected during the early time points to infect new cells. PBMCs were infected with supernatant collected at the different times and GFP-EBV was monitored by GFP expression using a fluorescence microscope as well as FACS to 7 days (Figure 8A and B, left panel). After 24 hours post-infection, GFP signals and clumping of the primary B-cells were visualized. FACS analysis also showed that 5% of the cells were GFP positive by 12 hours and increased to 20% after 72 hrs post infection (Figure 8A and 8B). Importantly, we showed that the lytic genes were expressed at a higher level when compared to latent genes suggesting a burst of lytic infection and particle release. To examine whether the lytic replication cycle produces virion particles released into the supernatant at each time point post infection, the supernatant was collected and used to infect fresh PBMCs. The infection of fresh PBMCs was monitored for GFP expression. The result showed that the supernatant from 72 hours post infection was capable of infecting fresh PBMCs followed by GFP expression and cell clumping (Figure 8B, right panel). The percentage of GFP expressing cells as determined by FACS was 1–2% from 72 h to 168 h. This result strongly suggested a burst of lytic replication and release of virion particles during the initial stages of EBV infection. However, the number of infected cells was significantly higher when the supernatant from 7 days of post-infection was used to infect new cells.

### Production of progeny GFP-EBV particles during early infection is inhibited by acyclovir

**Figure 9:**
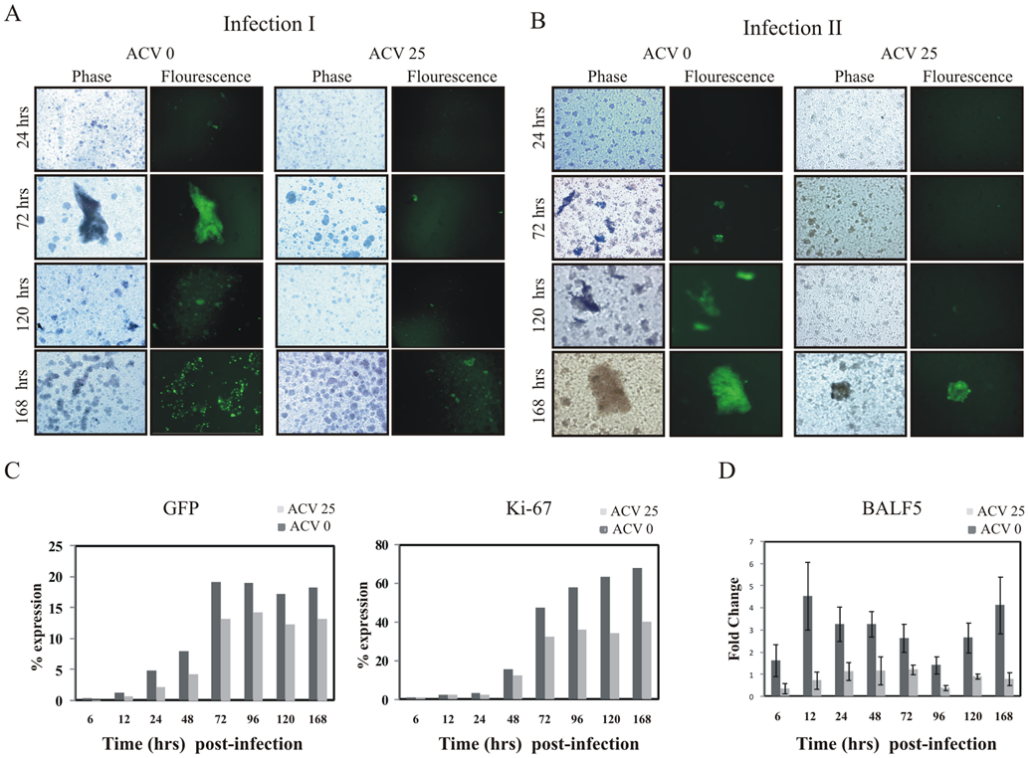
The progeny virus produced in the primary infection is inhibited by acyclovir. PBMCs were infected with GFP-EBV in presence and absence of 25 µM of ACV (infection-I) and at specific times postinfection the supernatant was collected and used infect fresh PBMCs cells (infection II). (A) Phase-contrast (left) and fluorescence (right) images of GFP-EBV infected PBMC cells are shown after specific times postinfection (24 hrs, 72hrs, 120 hrs and 168 hours) in absence of ACV (left panel) and in presence of 25 µM ACV (right panel). (B) Phase-contrast (left) and fluorescence (right) images of PBMC cells infected with supernatant from the above mentioned times post-infection from 24hrs, 72hrs, 120hrs and 168h (infection-II) in absence (left panel) and presence of 25 µM ACV (right panel). (C) Flow cytometry analysis of GFP (left panel) and Ki-67(right panel) expression at post-infection of different time intervals (6h, 12h, 24h, 48h, 72h, 96h, 120h and 168h) in absence and presence of 25 µM ACV. D. DNA polymerase BALF5 mRNA was also examined by qReal Time PCR after GFP-EBV infection at similiar intervals stated above in absence and presence of 25 µM ACV. To determine quality of the RNA, GAPDH mRNA was also amplified by RT-PCR. The fold change was calculated by the ΔΔCt method. Each data point shown is the average of three identical experiments. ± SD was shown in error bar.

To investigate lytic gene expression as well as the release of virion particles during early stages of infection, we monitored GFP-EBV infection to PBMCs in the presence of 25 µM ACV. PBMCs were infected with GFP-EBV with and without 25 µM ACV at the different times post-infection and infection was monitored by GFP expression using a fluorescence microscope as well as FACS analysis up to 7 days (Figure 9A, left panel and 9C). After 24 hours post-infection, GFP signals and clumping of the primary B-cells were clearly observed. FACS analysis also showed that 5% of the cells were GFP positive by 24 hours and increased to 20% after 72 hrs post infection, whereas in the presence of 25 µM ACV, the extent of GFP expression decreased (Figure 9A, right panel and 9B). It was observed that GFP expression was 13–15% in presence of ACV, whereas in absence of ACV it was ∼20%. The proliferation marker Ki-67 was also measured by flow cytometry in presence of ACV (Figure 9, right panel). The results showed that in the presence of 25 µM ACV, the expression of Ki-67 decreased by 1.5 fold. To test the inhibition effect of ACV on viral DNA polymerase synthesis, we checked the level of viral DNA polymerase (BALF5) gene expression by Real Time PCR in the presence of ACV shown in Figure 9D. The results clearly showed that in the presence of 25 µM ACV, the expression of BALF5 mRNA decreased by 4–5 fold decreased compared to the absence of ACV. These results suggest that in the presence of ACV, the rate of lytic replication is inhibited which results in lower infection of GFP-EBV and B-cell proliferation.

To examine the effect of ACV on the lytic replication cycle as well as the production of virion particles released into the supernatant at each time point postinfection, the supernatants from infection-I were collected and used to infect fresh PBMCs (infection-II). Infection-II was monitored by visualization GFP using fluorescence microscopy (Figure 9B). The GFP results showed that the release of virion particle was inhibited in presence of ACV at Infection-I. This data strongly supports the conclusion that virion particles produced due to burst of lytic replication during early stages of infection.

**Figure 10:**
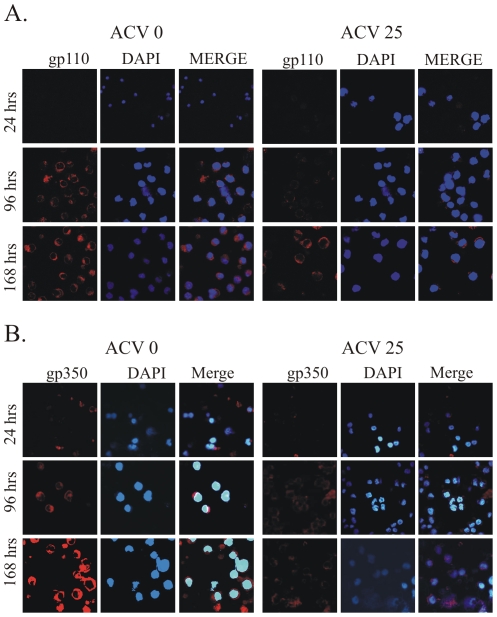
Glycoprotein expression during early stage of infection in presence of acyclovir. Endogenous expression of (A) gp110 and (B) gp350 were detected using mouse monoclonal antibody (1∶200 dilution), and rabbit respectively (1∶250 dilution). Primary antibodies were diluted in blocking buffer and incubated with fixed cells for 1 h at RT. Slides were washed three times (5 min each) with PBS and incubated with appropriate secondary antibody (1∶2000) for 1 h at RT followed by three times washes (5 min each) with PBS. The last wash contained 4′, 6′-diamidino-2-phenylindole (DAPI; Promega Inc., Madison, WI) for nuclear staining. Goat anti-mouse antibody Alexa Fluor 594 and goat anti-rabbit antibody Alexa Fluor 594 were purchased from Molecular Probes Inc. (Carlsbad, CA). Slides were then washed in PBS and mounted using Prolong anti-fade (Molecular Probes Inc, Carlsbad, CA). Fluorescence was viewed by confocal microscopy and analyzed with Fluoview 300 software from Olympus Inc. (Melville, NY). The images were sequentially captured using an Olympus confocal microscope. All panels are representative pictures from similar repeat experiments.

To support the above results that lytic replication occurred in EBV infected cells , we checked the expression of late genes glycoproteins gp110 and gp350 by detection of the protein sing fluorescence microscopy. PBMCs were infected with GFP-EBV. The expressions of glycoprotein (gp110 and gp350) were monitored by immunoflourescence analysis in the presence and absence of ACV at different times post-infection (Figure 10A and 10B). The results showed that in absence of ACV gp110 was expressed at 96 h whereas in presence of ACV, the gp110 expression was dramatically decreased. At 168h post-infection, the level of gp110 expression was higher in the absence of ACV as compared to the levels in the presence of ACV. The same result was also seen for gp350 expression (Figure 10B). Thus, the late protein expression profiles in ACV suggested that the productive cycle was inhibited by acyclovir and further supportive our conclusions that the bursts of replication and virion particle production during the early stages of infection is likely to be crucial for establishment of latency and transformation of the infected primary B-cells.

## Discussion

Using the BAC system, the viral genome can be propagated in *Escherichia coli*, and mutations can be rapidly and precisely introduced into any of the viral genes. To facilitate the generation of recombinant viruses, the EBV genome was first cloned into the bacterial artificial chromosome (BAC) [28], [29]. The resultant BAC based EBV genome was able to make virus and was capable of B-cell immortalization [29]. To monitor infection of primary B-cell by EBV, we introduced the GFP ORF into the EBV BAC by homologous recombination and the resultant construct was designated as BAC GFP-EBV [29]. The construction of BAC GFP-EBV was confirmed by exhaustive restriction enzyme digestion pattern, PCR analysis of the junctions, selected regions of EBV as well as southern blot analysis. In addition, the new BAC GFP-EBV clone was also competent for virus replication and B-cell immortalization. The induction of 293T cells stably maintaining the BAC GFP-EBV by chemical inducers produced progeny recombinant virus which was used to infect primary B-cells leading to the generation of LCLs expressing GFP. These studies strongly suggested that the recombinant GFP-EBV had similar infectious properties when compared to the wild type EBV. The advantage of this GFP expressing BAC-EBV system is that EBV infection and propagation in mammalian cells can now be monitored from the early stages post-infection to transformation of B-cells and generation of LCLs.

The infection of human B lymphocytes by EBV *in vitro* results in immortalization of the infected cells and augmentation of numerous B-cell surface antigens[65]. We wanted to obtain a more detailed picture of the early events after EBV infection and to monitor changes in expression of cell surface markers as a result of infection. We used infection of PBMCs by GFP-EBV to monitor the early stages post-infection. We used a panel of surface antigen markers CD5, CD10, CD19, CD23, CD39, CD40 & CD44 and the intracellular marker Ki-67 to correlate B-cell activation with proliferation during the early stages of infection up to 7 days. GFP positive cells were evaluated for expression of the surface proteins indicated. CD19, a specific surface antigen marker for B-cells [66], [67], was detected in greater than 90% of the GFP positive cells. This strongly suggested that the GFP-EBV infected cells were predominantly B-cells among the mixed population of PBMCs.

The cell surface protein CD5 expression on B cell can be up-regulated by a number of agents which results in B-cell activation [35]. CD5 has been shown to be important for apoptosis of antigen-receptor induced B lymphocytes [68]. The expression of CD5 was also shown to be regulated by EBV [37]. CD5 expression was suppressed in EBV transformed cells suggesting that that the virus may down-regulate its expression to prevent apoptosis of the transformed cell. Our data during the early stages of EBV infection showed that CD5 levels increased from 6 hrs to 5 days but was depressed after 5 days to relatively low levels seen in LCLs. Our data supports previous studies monitoring CD5 levels in transformed B-cell [37]. However, during the initial days of EBV infection (up to 5 days), the increased expression of CD5 is likely to be due to signaling as a result of virus-host interaction and resulting B-cells activation. Also, the infection of primary B-cell by EBV leads to killing of a large percentage of cells due to lytic replication during the early stage of infection. This resulting cell death may also be a reason for up-regulation of CD5 expression during the early stages of infection. CD10, another cell surface protein, preferentially expressed in germinal center is an activation marker for B-cells as the germinal center is the site for activation and proliferation of B-cells [69]. The expression of CD10 was unchanged after 6 hrs post-infection during the course of early infection of EBV. This suggests that the initial interaction between EBV and B-cells led to a rapid change in CD10 expression, which was maintained after 6 hrs followed by activation and cell proliferation.

The B-cell activation markers CD23, CD40 and CD44 have been shown to be associated with EBV infection [13]. Additionally, the viral proteins EBNA-2 and LMP-1 cooperatively induce the cell surface protein, CD23 [13]. CD23 expression induced by infection of B-cell with GFP-EBV showed an interesting trend in that CD23 expression increased and reached maximum levels by 72 hrs but then dramatically decreased by 7 days. However, EBNA-2 was expressed at the initial stage of early infection and reached maximum within 24 hours. These results suggest that the expression of EBNA-2 at the initial stage activates the CD23 expression which continued to increase up to 72 hrs. In addition, LMP-1 expression was detected after 48 hours which is also known to up-regulate CD23 expression [70]. Moreover, the increase in lytic replication by EBV during the early stages post infection is also expected to lead to cell death. This provides a possible explanation for the observed down-regulation of CD23 after 72 hrs. However, other possibilities exist in that additional latent antigens may also contribute to CD23 regulation and that the early increase in CD23 levels are important for persistence or signaling events that eventually leads to cell proliferation and transformation. The Cell surface protein CD40 is mainly expressed in antigen presenting cells, and plays a critical role in B-cell activation by providing cell survival signals via interaction with the CD40 ligand (CD40L) expressed on the surface of activated T-cells [71]. It is reported that signaling CD40 and its ligand CD40L contributes and is likely to be critical for the antiapoptotic function of EBV and B cell transformation in the presence of LMP1 after EBV infection [72]. Our results showed that the expression level of CD40 from GFP positive cells (infected cells) increased from 6 hrs post-infection and continued to increase up to 7 days, and are maximally expressed in transformed EBV positive LCLs. The increased expression of CD40 due to EBV infection also activates the CD40 signaling pathway which suppresses apoptosis and promotes proliferation of infected cells [73]. CD44 is a cell adhesion molecule which exists in multiple isoforms associated with tumorigenesis and metastasis [74]. The level of CD44 was detected by 6 hrs post-infection and was maintained throughout our study suggesting an important contribution to the proliferation and transformation process.

Ki-67 is a nuclear antigen that is expressed in proliferating cells during the different phases of the cell cycle and its expression is used as a marker for cell proliferation [34]. It is reported that Ki-67 is expressed in CD19 positive cells in B-CLL [42]. It was also observed that Ki-67 positive cells infected with EBV express EBNA2 [42]. We showed that expression of Ki-67 post-infection of B-cell by GFP-EBV was detected by 48 hrs and consistently increased after 7 days as well as in EBV transformed cells. Therefore, B-cell proliferation is most likely initiated a few hours after EBV infection eventually leading to B-cell transformation and LCLs. The delayed expression of Ki-67 (at 48 hours) is likely to be due to lytic replication of infected cells at the early stages of infection where the infected cells may not survive but produces progeny capable of infecting new cells that eventually persists and establishes latency. This result strongly supports the hypothesis that during the early stages of EBV infection primary B-cells undergo lytic replication important for persistence of the virus, latency and transformation to LCLs. It is also possible that the lytic genes may contribute and is important to the upregulation of cellular genes important for driving cell proliferation and transformation.

In an effort to understand the latent and lytic gene expression profile during early stage of EBV infection we used semi-quantitative real time PCR to determine the levels of transcript for the latent genes EBNA-1, EBNA-2 and LMP-1 [7] as well as the immediate early, early and late lytic genes BZLF1, BALF5 and BcLF1 [14]. Data from the Real time PCR showed that the latent genes EBNA-1, EBNA-2 and LMP-1 were expressed along with the lytic genes BZLF1, BALF5 and BcLF1.

Alfieri *et al.* [75] prevoiusly showed latent gene expression by immunostainig during initial stages of infection (up to 3 days) of PBMCs by EBV. They showed that EBNA-1 expressed at 20–32 hrs post-infection and reached levels seen in LCLs at 46–70 hrs post-infection, whereas EBNA-2 expressed at 16 hrs post-infection and LMP-1 expressed in 48 h post-infection. Expression of EBNA-1 and EBNA-2 was similiar in our studies. However, we observed that LMP-1 was expressed at an earlier time post-infection (maximum at 24 hours) when compared to the previous report (maximum at 48 hours) [75]. However, Yuan et al. [76] reported that induction of lytic infection by IgG crosslinking, EBNA and LMP mRNA were expressed which supports our result. Since the gene expression profile of our results suggests that after infection with GFP-EBV, PBMCs establishes latent infection as well as lytic replication at during the early stages of post-infection. As infected cells are undergoing latent and lytic replication at the same time and are in the overall cell population, it would be difficult to measure the precise number of cells in the lytic or latent phase of infection and the cells expressing LMP-1 during the early stages of infection. Further, studies are ongoing to determine whether or not the expression patterns we see are directly related to latent or lytic replication.

Since BZLF1 was strongly expressed during the early phase of infection the lytic genes are then induced resulting in progeny and infection of new cells. Expression of the DNA polymerase (BALF5) and major capsid protein BcLF1, also indicate that virion particles were produced which was confirmed by infection of fresh PBMCs using supernatant from primary infection. The infection of fresh PBMCs strongly showed that virion particles were produced during the early stages of infection (Figure 8). To determine if the production of virion particles occurred during the early stages of infection, we used acyclovir in the course of infection. The inhibition of viral DNA polymerase expression as well as the late lytic protein (gp110 and gp350) expression due to addition of ACV strongly supported our conclusion that virion particles produced were due lytic replication and not from virus produced on from the initial infection (see Figure 9 and Figure 10). Additionally, the insertion of the cassette in the B95-8 deletion site was the least deleterious in affecting changes in lytic replication as BZLF1 is located at the distant position in the genome from the insertion site and that induction of the other late genes are dependent on BZLF1 expression. Thus, we were confident that the early lytic replication is important to contributing to establishment of latency and transformation of primary infected B-cells. Additionally, since latent genes were also produced during the early infection, there is most likely a finely tuned mechanism for early induction of lytic genes and viral progeny production important for triggering cell proliferation and a switch from a lytic type infection to a latent infection in the newly infected cells.

**Figure 11:**
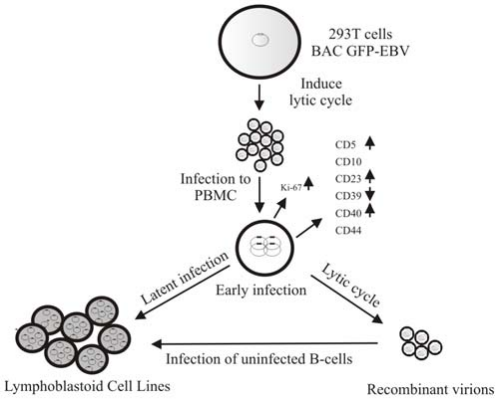
Schematic representation of events during early infection of PBMCs by GFP-EBV leading to B cell transformation.

LCL were initially believed to arise from direct outgrowth of *in vivo*-infected EBV–carying B-cells (One step mechanism)[77]. However, Rickinson et al. [78], [79] showed that cell lines can also be generated in two steps: release of virus from infected cells during the initial period of *in vitro* cultivation, followed by the secondary immortalization of normal B-cells in vitro. Lewin *et al*.[80] showed experimentally that the 2-step mechanism is more common. Our results also suggest that the 2-step mechanism (the virus particles, due to productive cycle at the early stage of infection, competent for infection of uninfected B-cells and immortalization of B-cells) is the most probable for infected primary B-cells to be driven to transformation by EBV infection *in vitro* (see Figure 11). However, studies are ongoing to carefully address these questions and will provide a more detailed molecular mechanism of this process. Additionlly, the induction of lytic genes may also be committed to a small population of the infected cells that eventually dies but are critical for induction of the proliferative genes and switching to a latent type infection. It would certainly be important to determine whether the infected cells can undergo same level of lytic replication, survive and is eventually switch to latent infection and transformation or whether only a committed number of cells in the population goes lytic replication, dies but is critical for the remainder of the population to survive and B transformed.
