# Expression of Concern: Early Events Associated with Infection of Epstein-Barr Virus Infection of Primary B-Cells

**DOI:** 10.1371/journal.pone.0256674

**Published:** 2021-08-19

**Authors:** The PLOS ONE Editors

Following the publication of this article [1], concerns were raised regarding results presented in Figs 3, 9, and 10. Specifically,

- In Fig 3A, irregularities have been detected in the background signal surrounding bands in the following lanes:
      ○ BamHI T panel: lanes 1–7
      ○ BamHI K panel: lanes 2–7
      ○ BamHI E panel: lane 7
      ○ BamHI H panel: lanes 3–7
      ○ BamHI C panel: lanes 1, 3, 5, 7
      ○ Puro panel: lanes 3, 5, 6
      ○ EGFP panel: lanes 1, 5, 7
- In Fig 9B, the ACV25 24hrs Fluorescence panel appears similar to the ACV25 72 hrs Fluorescence panel.
- In Fig 10A, the ACV 0 96 hrs DAPI panel appears similar to the ACV 25 96 hrs DAPI panel.

The authors clarified that the Fig 9B ACV25 24hour panel and the Fig 10A ACV 0 (96h) DAPI panel were inadvertently duplicated during figure preparation and provided the updated Figs 9 and 10 below to correct the duplicated panels. Furthermore, the authors indicated that the irregularities in the Fig 3A results are due to the resolution of the camera, dust or particulate matter on the gel, and the way the images were captured in the gel documentation system. They explained that raw data underlying the published PCR results are no longer available due to the Gel Doc system having been replaced in the time since the experiments were conducted. In the absence of the original data underlying the Fig 3A results, the concerns cannot be resolved.

**Fig 9:**
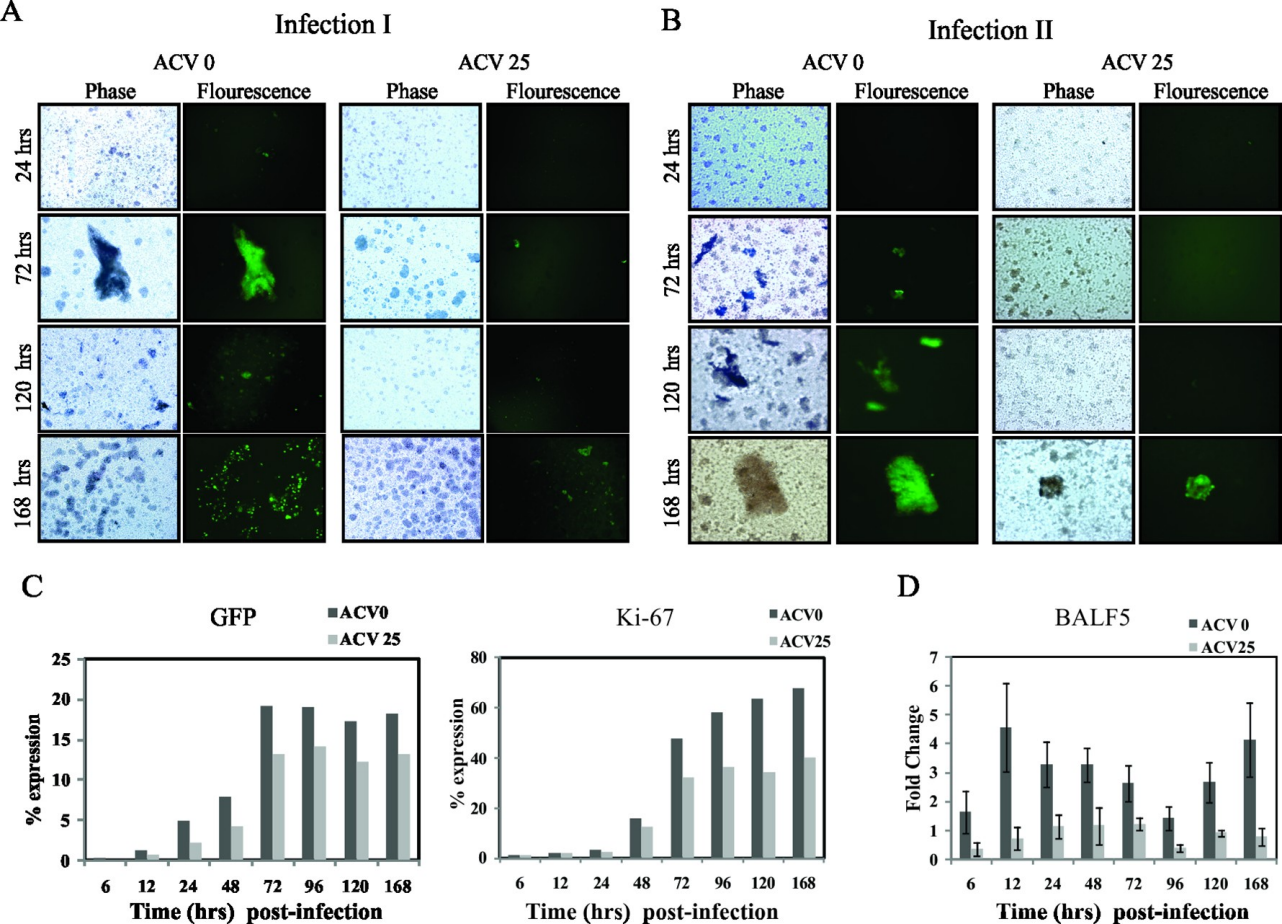
The progeny virus produced in the primary infection is inhibited by acyclovir. PBMCs were infected with GFP-EBV in presence and absence of 25 µM of ACV (infection-I) and at specific times postinfection the supernatant was collected and used infect fresh PBMCs cells (infection II). **(A)** Phase-contrast (left) and fluorescence (right) images of GFP-EBV infected PBMC cells are shown after specific times postinfection (24 hrs, 72hrs, 120 hrs and 168 hours) in absence of ACV (left panel) and in presence of 25 µM ACV (right panel). **(B)** Phase-contrast (left) and fluorescence (right) images of PBMC cells infected with supernatant from the above-mentioned times post-infection from 24hrs, 72hrs, 120hrs and 168h (infection-II) in absence (left panel) and presence of 25 µM ACV (right panel). **(C)** Flow cytometry analysis of GFP (left panel) and Ki-67(right panel) expression at post-infection of different time intervals (6h, 12h, 24h, 48h, 72h, 96h, 120h and 168h) in absence and presence of 25 µM ACV. **(D)** DNA polymerase BALF5 mRNA was also examined by qReal Time PCR after GFP-EBV infection at similiar intervals stated above in absence and presence of 25 µM ACV. To determine quality of the RNA, GAPDH mRNA was also amplified by RT-PCR. The fold change was calculated by the ΔΔCt method. Each data point shown is the average of three identical experiments. ± SD was shown in error bar.

**Fig 10:**
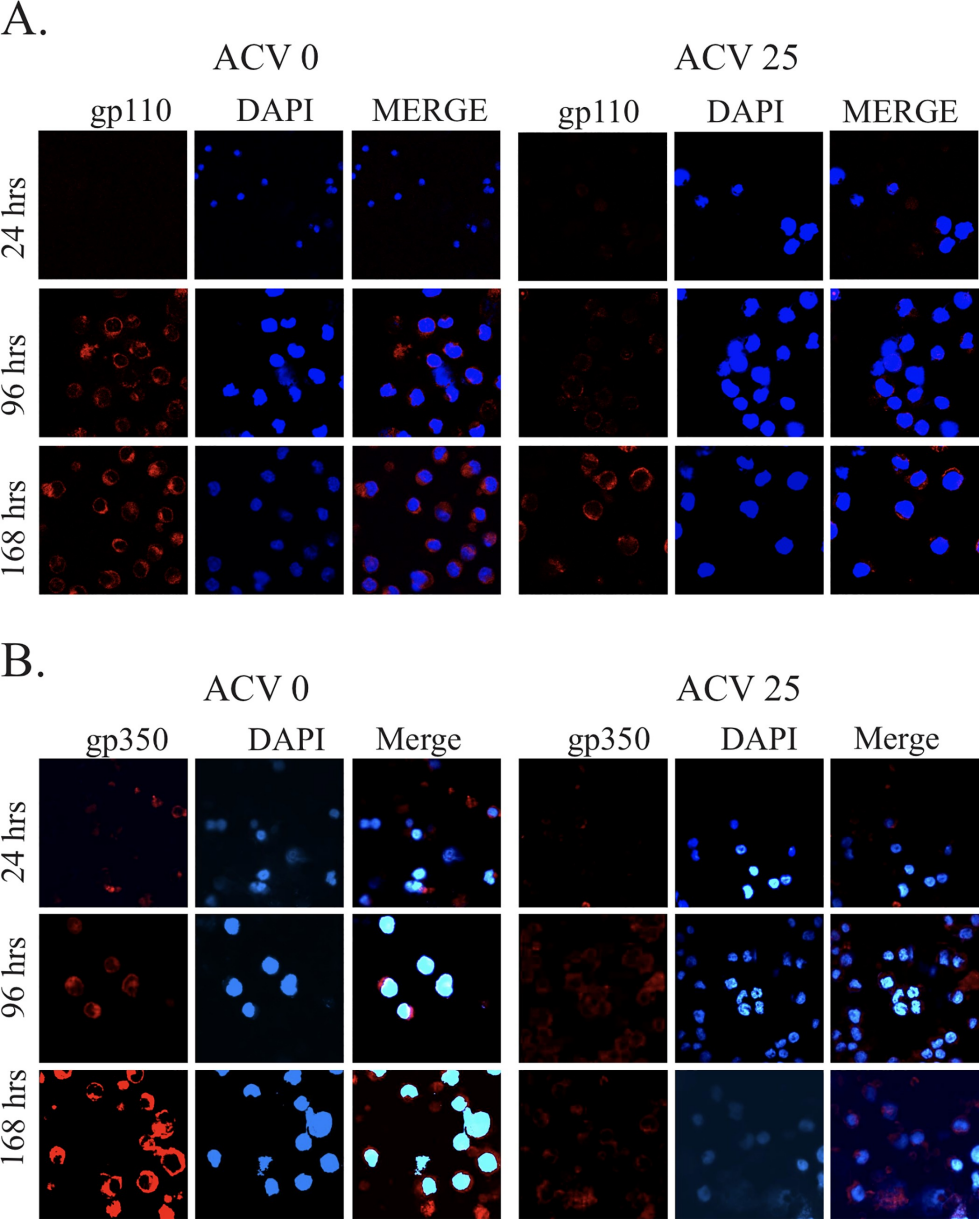
Glycoprotein expression during early stage of infection in presence of acyclovir. Endogenous expression of **(A)** gp110 and **(B)** gp350 were detected using mouse monoclonal antibody (1∶200 dilution), and rabbit respectively (1∶250 dilution). Primary antibodies were diluted in blocking buffer and incubated with fixed cells for 1 h at RT. Slides were washed three times (5 min each) with PBS and incubated with appropriate secondary antibody (1∶2000) for 1 h at RT followed by three times washes (5 min each) with PBS. The last wash contained 4′, 6′-diamidino-2-phenylindole (DAPI; Promega Inc., Madison, WI) for nuclear staining. Goat anti-mouse antibody Alexa Fluor 594 and goat anti-rabbit antibody Alexa Fluor 594 were purchased from Molecular Probes Inc. (Carlsbad, CA). Slides were then washed in PBS and mounted using Prolong anti-fade (Molecular Probes Inc, Carlsbad, CA). Fluorescence was viewed by confocal microscopy and analyzed with Fluoview 300 software from Olympus Inc. (Melville, NY). The images were sequentially captured using an Olympus confocal microscope. All panels are representative pictures from similar repeat experiments.

The original data underlying the results presented in Figs 3A and 9A are no longer available. The individual level data underlying Fig 9C and 9D are provided in the S1 and S2 Files below. The data underlying the remaining results presented in this article are available from the corresponding author upon request.

The *PLOS ONE* Editors issue this Expression of Concern to notify readers of the above concerns and relay the supporting data and updated figures provided by the corresponding author.

## Supporting information

S1 File Data underlying Fig 9C. (XLSX)

S2 File Data underlying Fig 9D. (XLS)
